# Evaluation of the Validity and Reliability of the Persian Version of IBDcQ-8 Questionnaire

**DOI:** 10.34172/aim.36812

**Published:** 2026-02-01

**Authors:** Setareh Moallem, Masoud M Malekzadeh, Amir Kasaeian, Sudabeh Alatab, Alireza Sima, Mitra Ahadi, Ali Bahari, Homayoun Vahedi, Reza Malekzadeh

**Affiliations:** ^1^Department of Gastroenterology and Hepatology, Faculty of Medicine, Mashhad University of Medical Sciences, Mashhad, Iran; ^2^Digestive Disease Research Center, Digestive Disease Institute, Tehran University of Medical Sciences, Tehran, Iran; ^3^Liver and Pancreato-biliary Diseases Research Center, Digestive Diseases Research Institute, Tehran University of Medical Sciences, Tehran, Iran; ^4^Digestive Oncology Research Center, Digestive Diseases Research Institute, Tehran University of Medical Sciences, Tehran, Iran; ^5^Research Center for Chronic Inflammatory Diseases, Tehran University of Medical Sciences, Tehran, Iran; ^6^Sasan Alborz Biomedical Research Center, Masoud Gastroenterology and Hepatology Center, Tehran, Iran

**Keywords:** IBD control questionnaire, Persian, Validity and reliability

## Abstract

**Introduction:**

The 8-item Inflammatory Bowel Disease control Questionnaire-8 (IBDcQ-8) is a patient-reported measure designed to capture the core domains of disease control and quality of life. The aim of this study was to evaluate the validity and reliability of the Persian version of the IBDcQ-8 in the Iranian IBD population.

**Methods:**

A standardized forward–backward translation procedure was employed to adapt the questionnaire into Persian. Content validity was evaluated through structured interviews with a panel of 10 evaluators. Construct validity was examined by structured interview and clinical visits of 101 patients. Harvey Bradshaw Index (HBI), partial Mayo score, and IBD Questionnaire (IBDQ) were used as comparator instruments. Patients’ responses were compared with clinical global assessment, Harvey Bradshaw Index (HBI), partial Mayo score, IBDQ score, and laboratory markers using Spearman’s rank correlation. Test–retest reliability was evaluated by calculating the intraclass correlation coefficient (ICC) between the two subsequent interviews. A *P*-value<0.05 was considered statistically significant.

**Results:**

Half of the subjects (n=51 (50.5%)) were female. The mean age was 38 years (SD=14). Sixty-seven patients (66%) had UC. IBDcQ-8 scores showed strong correlations with HBI and IBDQ, and moderate correlations with the partial Mayo score, CRP, and clinical evaluation at the first visit (*P*-value<0.05). Agreement was excellent for one-day follow-up (ICC=0.82, 95% CI: 0.71–0.89, *P*<0.001), and moderate for two-week follow-up (ICC=0.61, 95% CI: 0.44–0.73, *P*<0.001).

**Conclusion:**

The Persian version of the IBDcQ-8 questionnaire demonstrated robust validity and acceptable reliability even when administered by telephone interview, supporting its usefulness for evaluating disease-specific quality of life in patients with IBD and its applicability in future studies.

## Introduction

 Inflammatory Bowel Disease (IBD), including Crohn’s Disease (CD) and Ulcerative Colitis (UC), refers to chronic inflammatory disorder affecting the gastrointestinal system. Since IBD is characterized by periods of exacerbation and remission and is also chronic with persistent activity, there has been a growing attention to Quality of Life (QoL) among patients suffering from this disease.^1^

 In recent years, various tools have been developed to assess QoL that examine multiple domains, including physical, emotional, social, and aspects of daily functioning.^2-4^ These questionnaires are extensively utilized in clinical visits, as well as in clinical trials and epidemiological studies. However, due to the often lengthy and time-consuming nature of many of these instruments, short-form questionnaires may be more practical for use in research studies and clinical appointments.^3,5-7^

 Several disease-specific instruments have been developed to evaluate Health Related Quality of Life (HRQoL) in individuals with IBD. Among them, the Inflammatory Bowel Disease Questionnaire (IBDQ) is one of the most widely used tools, available in various versions differing in length and complexity since its introduction in 1989.^8^ While the original IBDQ includes 32 items, shorter versions have been introduced to facilitate use in busy clinical settings without compromising measurement precision.^9^ This questionnaire was translated to Persian and validated by Maleki *et al.*^10^

 The IBD-Control questionnaire, developed by Bodger *et al.* in 2014, is a rapid, patient-reported measure designed to capture disease control and HRQoL using a recall period of two weeks.^11^ The Inflammatory Bowel Disease control Questionnaire-8 (IBDcQ-8) consisting of 8 items, is a concise, patient-reported measure designed to capture the core domains of disease control and quality of life consisting of symptoms, daily functioning, and well-being.^7,11^ Due to its brevity and ease of administration, it holds promise for both clinical practice and research. However, before being implemented in a new cultural or linguistic context, it is necessary to establish its psychometric properties, including validity and reliability.

 Several studies have indicated that the prevalence and incidence of inflammatory bowel disease in Iran are on the rise.^12^ Given the chronic nature of this disease and the significant costs associated with its treatment, and its impact on patients’ quality of life, a registry system for inflammatory bowel disease has been designed and implemented to facilitate better understanding of the condition within the country and improving patient management and conducting research. The Iranian Inflammatory Bowel Disease Registry (IRCC) commenced operations four years ago and has recorded over 10,000 patients within this registry. A pilot study has been conducted on this database, and the validity and reliability of its questionnaire has been preliminarily examined.^13^

 Within this registry, the IBDcQ-8 is employed as the HRQoL assessment tool for enrolled patients. Given the importance of ensuring the trustworthiness of registry data, the aim of this study was to evaluate the validity and reliability of the Persian version of the IBDcQ-8 in the Iranian IBD population, providing evidence to support its use in both clinical practice and research.

## Materials and Methods

###  Definition

 Evaluation of validation refers to the extent to which the questions in a questionnaire correspond to the intended reality. This evaluation is conducted across various dimensions. The first aspect pertains to the item level, known as content validity, which is also referred to as face validity. At a broader level, the evaluation encompasses the overall structure of the questionnaire, its domains, and the relationships and distinctions among its components, in comparison with other standard criteria, a concept referred to as construct validity.^14,15^

 Reliability assessment pertains to the reproducibility of the study results. This can be evaluated through examining the interrelationships among the components of the questionnaire (internal consistency) or by conducting the test twice within a short interval (2 to 4 weeks), a method known as test-retest reliability.^15^ However, there are other types of reliability that are not relevant to our study.

###  Participants

 In this study, a sample size of 101 patients was enrolled, which satisfies the recommended item-to-response ratio of 10:1 for psychometric studies. Patients with a confirmed diagnosis of IBD, who presented to the outpatient clinics of two gastroenterologists cooperating with IRCC, were consecutively enrolled. They were invited to participate and, after providing informed consent, were interviewed by trained research personnel for the administration of the IBDcQ-8 in order to evaluate disease-specific quality of life in all participants. This study was approved by research ethic committee of Digestive Disease Research Institute, Tehran University of Medical Sciences in keeping with the Declaration of Helsinki (approval number: IR.TUMS.DDRI.REC.1401.005).

###  Questionnaire

**Figure 1:**
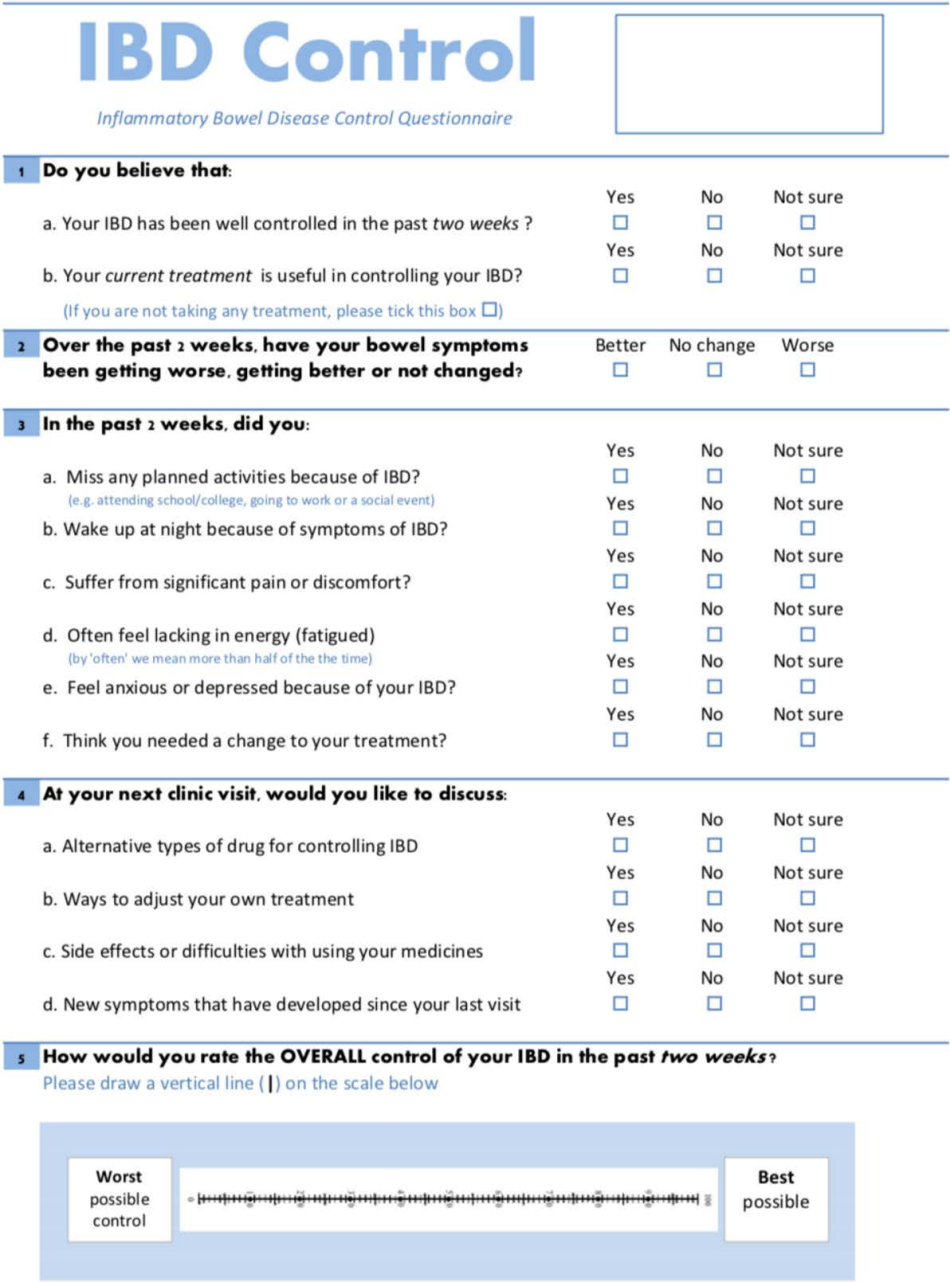
IBDcQ-8 Questionnaire

 The IBDcQ-8 questionnaire, a validated short form of the 13-item IBDcQ, was employed to assess disease activity and the higher the score, the lower the disease activity. It was originally developed in the United Kingdom, and its processes of item reduction, validation, and reliability testing have been previously reported.^11^ The IBDcQ-8 consists of eight items, derived specifically from item set 1 and item set 3 of the parent questionnaire (Figure 1).

 Each item is rated on a three-point scale ranging from 0 to 2, where higher scores reflect better health status and lower symptom burden. The total score is obtained by summing all eight items, yielding a possible range of 0 to 16. Based on previously established thresholds, a cumulative score of > 13 is indicative of disease remission, whereas scores of ≤ 13 denote active disease.

###  Assessment of Content Validity 

 A standardized forward–backward translation procedure was employed to adapt this internationally validated questionnaire into Persian. Initially, the questionnaire was translated into Persian by a professional translator with expertise in medical terminology. The translated version was then independently back-translated into English by a another bilingual physician. Discrepancies between the original and back-translated versions were precisely reviewed, and modifications were implemented to ensure semantic, idiomatic, and conceptual equivalence. Content validity was subsequently evaluated with respect to relevance and clarity through structured interviews, conducted either in-person or via virtual meetings, with a panel of 10 evaluators. This panel included six gastroenterologists (Dr. Malekzadeh, Dr. Vahedi, Dr. Sima, Dr. Naserimoghadam, Dr. Merat, and Dr. Pourshams), two experts in biostatistics and epidemiology (Dr. Kasaeian and Dr. Etemadi), and two patients with the target condition. All evaluations were documented using the standardized content validity assessment form (Supplementary File, Table S1).

###  Study Protocol for Assessing Construct Validity and Reliability

 To assess construct validity, 101 patients whose data had recently been entered into the national IBD registry were consecutively enrolled using a convenience sampling approach. Prior to entering the consultation room, each patient completed the questionnaire during a face-to-face interview with a trained interviewer. Immediately thereafter, the patient underwent examination by a gastroenterologist, who recorded a global assessment of disease activity for both the preceding two weeks and the preceding six months using two structured three-point questions. Concurrently, relevant laboratory parameters (including Complete Blood Count (CBC), Hemoglobin (Hb), C-Reactive Protein (CRP), and Fecal Calprotectin (FCP)) were documented in a separate form by the interviewer.

 IBDcQ-8 is designed for self-administration but registry data are routinely collected through structured telephone interviews.^13^ To evaluate the reliability and validity of this telephone interview, patients who were enrolled in study were identified three days in advance of their clinical visit. Using randomized block allocation (blocks of four), some patients were contacted by telephone the day before their clinic visit, and others the day after, and the questionnaire was filled by trained interviewers during telephone interview. The results were then compared with the in-person responses.

**Figure 2:**
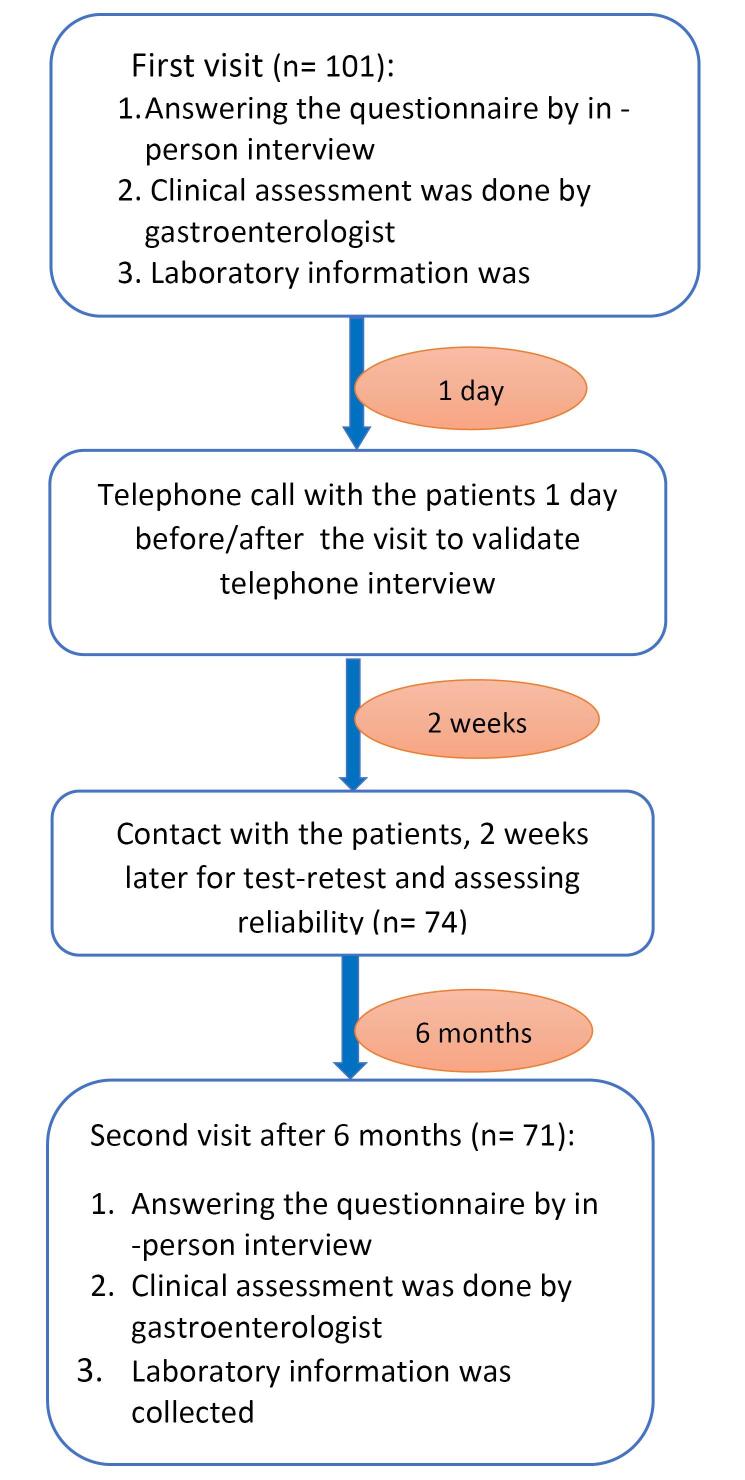
Study Protocol for Evaluation of Construct Validity and Reliability of IBDc8 Questionnaire

 Two weeks after the initial visit, patients were contacted again by telephone, and the questionnaire was re-administered for comparison with baseline in-person data. Finally, at the six-month follow-up visit, patients first completed the questionnaire in person, followed by the physician’s clinical assessment, and the results were compared with baseline measurements to evaluate the stability of the construct over time (Figure 2).

 Responses and total scores during face-to-face and telephone interview were compared with three reference measures to assess correlation: (1) a gastroenterologist’s clinical assessment of disease activity based on symptoms, laboratory tests, endoscopic findings, and imaging; (2) laboratory parameters, including Hb, CRP, and FCP at diagnosis and within two weeks before the interview; and (3) standardized, interviewer-administered instruments comprising the Persian version of the IBDQ, the Harvey–Bradshaw Index (HBI) for CD, and the partial Mayo Score for UC.

###  Statistical Analysis

**Figure 3:**
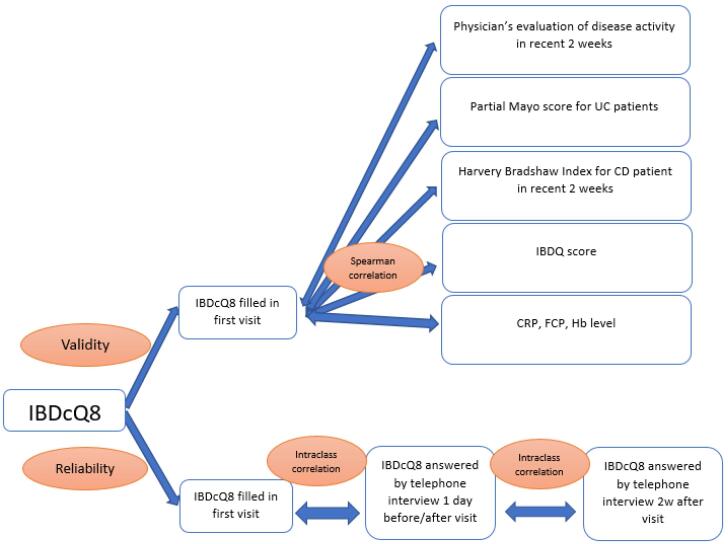
Statistical Analysis Process for Evaluation of Validity and Reliability of the IBDcQ8 Questionnaire

 Construct validity was examined by correlating patient-reported questionnaire responses with the gastroenterologist’s global assessment, HBI, partial Mayo score, IBDQ, and laboratory markers (CRP and FCP) using Spearman’s rank correlation, with coefficients interpreted according to Table 1. Differences in mean scores between categories were assessed with the Kruskal–Wallis test. Also, Cronbach’s alpha was calculated to check the internal consistency of translated questionnaire. Test–retest reliability was evaluated by calculating the intraclass correlation coefficient (ICC) between the two administrations. A *P*-value < 0.05 was considered statistically significant. The statistical analysis workflow is illustrated in Figure 3. Analysis was done with SATA version 16.

**Table 1:** Interpretation of Spearman R

**Spearman R**	**Correlation**
> 0.7	Very strong relationship
0.4-0.69	Strong relationship
0.3-0.39	Moderate relationship
0.2-0.29	Weak relationship
0.01-0.19	No or negligible relationship

## Results

 Of the 101 enrolled patient, 51 (50.5%) were female. The mean age was 38 years (SD = 14). Sixty-seven patients (66%) had UC, and the remainder had CD. According to the partial Mayo score, 43% of subjects with UC were in remission; 35%, 21%, and 2% had mild, moderate, and severe disease, respectively. For patient with CD, 57% were in the quiescent phase, and 43% had active disease (HBI score). Additional disease-related characteristics are presented in Table 2.

**Table 2:** Demographic Characteristics of Participants.

**Characteristics**	**First visit **N (%)	**Second visit **N (%)		**First visit **N (%)	**Second visit **N (%)
Gender			IBDcQ8		
Female	51 (50.5)	37 (52.22)	Quiescent ( > 13)	40 (39.60)	52 (73.24)
Male	50 (49.5)	34 (47.89)	Active ( = < 13)	61 (60.4)	19 (26.76)
Age			IBDMI		
< 18	4 (3.96)	2 (2.82)	Quiescent(5-6)	44 (44)	35 (49.30)
18 = < < 40	61 (60.4)	43 (60.56)	Active ( < 5)	56 (56)	36 (50.70)
40 = < < 60	26 (25.74)	19 (26.76)	IBDQ
= < 60	10 (9.9)	7 (9.86)	Quiescent ( = < 180)	83 (82.18)	28 (39.44)
Diagnosis			Active ( > 180)	18 (17.82)	43 (60.56)
UC	67 (66.34)	43 (60.56)	HBI
CD	34 (33.66)	28 (39.44)	Quiescent ( < 4)	58 (57.43)	57 (80.30)
CD phenotype			Active ( > = 4)	43 (42.57)	14 (19.72)
Non	12 (35.29)	8 (28.57)	Partial Mayo score
Fistulizing	6 (17.65)	5 (17.86)	Quiescent ( < 2)	43 (42.57)	52 (73.24)
Stricturing	6 (17.65)	5 (17.86)	Mild (2 = < < 5)	35 (34.65)	13 (18.31)
Both	4 (11.76)	4 (14.29)	Moderate (5 = < < 8)	21 (20.79)	5 (7.04)
missing	6 (17.65)	6 (21.43)	Severe ( > = 8)	2 (1.98)	1 (1.41)
CD extension			Clinical disease activity in 2 weeks		
Ileal	10 (29.41)	6 (21.43)	Quiescent	56 (55.45)	50 (73.53)
Colonic	13 (38.24)	12 (42.86)	Partially controlled	34 (33.66)	15 (22.06)
Ileo-Colonic	7 (20.59)	6 (21.43)	Active	11 (10.89)	3 (4.41)
Missing	4 (11.76)	4 (14.29)	Clinical disease activity in 6 months
UC extension			Quiescent	54 (53.5)	50 (73.5)
Proctitis	6 (9.23)	5 (11.63)	Partially controlled	34 (33.7)	15 (22.1)
Left Sided	19 (29.23)	14 (32.56)	Active	13 (12.9)	3 (4.4)
Pancolitis	40 (61.54)	24 (55.81)	Fecal calprotectin
Surgery due to IBD			Normal FCP	4 (3.96)	8 (7.92)
No	77 (77.78)	53 (80.30)	High FCP ( > 150mcg/g)	12 (11.88)	10 (9.90)
Yes	22 (22.22)	13 (19.70)	Missing	85 (84.16)	83 (82.18)

CD: Crohn’s disease, UC: ulcerative colitis, IBDcQ8: IBD control questionnaire, IBDMI: IBD Manitoba index, IBDQ IBD questionnaire, HBI: Harvey Bradshaw Index.

###  Validity

Table 3 summarizes the correlations between IBDcQ-8 scores at the first and second visits and the corresponding results of the HBI, partial Mayo score, full IBDQ, and laboratory parameters. As summarized in Table 3, IBDcQ-8 scores showed strong correlations with HBI and IBDQ, and moderate correlations with the partial Mayo score, CRP, and clinical evaluation at the first visit, all reaching statistical significance except FCP and Hb. At the second visit, significant correlations persisted with HBI, IBDQ, and partial Mayo, while correlations with clinical evaluation and other laboratory parameters were not significant.

**Table 3:** IBDcQ-8 Correlation Analysis at Two Time Points (Spearman’s R).

**Visit**	**HBI (P-value)**	**Partial Mayo** **(P-value)**	**IBDQ** **(P-value)**	**CRP** **(P-value)**	**FCP** **(P-value)**	**Hb** **(P-value)**	**Clinical evaluation** **(P-value)**
First visit	-0.63 (0.00)	-0.45 (0.00)	0.62 (0.00)	-0.42 (0.00)	-0.27 (0.11)	0.02 (0.82)	-0.39 (0.00)
Second visit	-0.50 (0.01)	-0.31 (0.0.4)	0.58 (0.00)	N/A	N/A	N/A	-0.13 (0.27)

CD: Crohn’s disease, UC: ulcerative colitis, IBDcQ8: IBD control questionnaire, IBDMI: IBD Manitoba index, IBDQ IBD questionnaire, HBI: Harvey Bradshaw Index.

**Figure 4:**
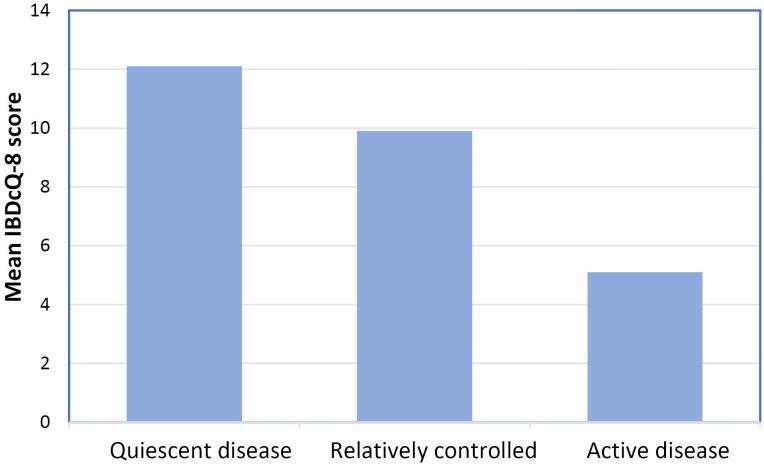
Mean IBDcQ-8 scores in Clinically Categorized Patients

 Differences in IBDcQ-8 scores among patients categorized by the physician as quiescent, relatively controlled, or having active disease were statistically significant (*P* < 0.001). Mean scores were 12.1 in the quiescent group, 9.9 in the relatively controlled group, and 5.1 in the active disease group, illustrating a consistent decline in IBDcQ-8 scores with increasing disease activity (Figure 4).

###  Reliability

 Cronbach’s alpha was 0.82 and showed high internal consistency in this instrument. Agreement between total IBDcQ-8 scores obtained at the initial visit, one day before or after visit, and at the two-week follow-up was assessed using the ICC. The result was excellent for one-day follow-up (ICC = 0.82, 95% CI: 0.71–0.89, *P* < 0.001), while agreement between baseline and the two-week follow-up was moderate (ICC = 0.61, 95% CI: 0.44–0.73, *P* < 0.001). Of note, the agreement between the one-day and the two-week follow up was higher than baseline agreement (ICC = 0.70, 95% CI: 0.52-0.82) (Table 4).

**Table 4:** Results of Inter Class Correlation (ICC) for the IBDcQ-8 Questionnaire.

	**First visit**	**Telephone interview 1day before/after**
	**ICC (95%CI)**	**ICC (95%CI)**
Telephone interview 1day before/after	0.82^*^ (0.71-0.89)	—
Telephone interview 2 weeks after	0.61^*^ (0.44- 0.73)	0.70^*^ (0.52-0.82)

CI: Confidence interval ^*^P-value < 0.05

## Discussion

 Assessing HRQoL has become an essential component of clinical evaluation and research in IBD, as traditional clinical measures alone cannot fully capture the patient’s perspective and disease burden. From another perspective, patient-reported information plays a pivotal role in clinical investigations. Imaging findings and laboratory parameters, while indispensable for objective disease assessment, do not necessarily capture the perceived severity of illness from the patient’s standpoint. It is often the subjective burden of symptoms that ultimately motivates patients to seek medical attention and adhere to therapeutic interventions. Moreover, patient self-reported measures are advantageous in that they are quick and simple to administer, cost-effective, and feasible for large-scale application, thereby offering an essential complement to conventional clinical and paraclinical evaluations.^16,17^

 Despite these advantages, the use of patient self-reported data is not without limitations. Subjective assessments are inherently vulnerable to recall bias, inter-patient variability, and the influence of psychological or social factors, which may compromise their reliability and reproducibility.^18,19^ Furthermore, patients’ interpretations of symptom severity can differ substantially depending on cultural background, health literacy, and coping mechanisms, thereby introducing heterogeneity into the collected data.^20^ In addition, self-reported outcomes may not always align with objective clinical or biomarker-based indices, which can complicate their integration into standardized disease activity measurements. Consequently, patient-reported data should be interpreted with caution and ideally in conjunction with objective clinical assessments to provide a more comprehensive evaluation of disease status.

 Standardized and validated patient-reported outcome (PRO) instruments are therefore crucial to ensure accurate assessment across different cultural and linguistic contexts. Shortened versions of QoL questionnaires, such as the IBDcQ-8, have been developed to balance comprehensiveness with feasibility in busy clinical settings. However, to be effectively implemented in diverse populations, these tools require rigorous translation and psychometric validation to confirm their reliability, validity, and cultural appropriateness.

 Recent studies have demonstrated good validity, reliability, and responsiveness of the IBDcQ in various countries and languages, including large-scale national surveys,^21^ cross-cultural translations and validation in various populations.^22-24^ These studies support its potential as a standardized, internationally applicable tool. However, to our knowledge, no validation study has yet been conducted in Iran.

 In the present study, the Persian version of the IBDcQ-8 was subjected to a comprehensive psychometric evaluation, with a particular focus on its validity and reliability (Supplementary File, Table S2). The findings of this study provide evidence for the content and construct validity of the Persian IBDcQ-8. As expected, the strongest correlation was observed with the full version of the IBDQ, since both instruments assess disease activity and quality of life over the preceding two weeks. In addition, the IBDcQ-8 demonstrated significant inverse correlations with the HBI in patients with CD and the partial Mayo score in patients with UC, reflecting differences in scoring direction: higher scores in the IBDcQ-8 and IBDQ indicate better quality of life and lower disease activity, whereas higher HBI and partial Mayo scores represent more active disease. Similarly, the questionnaire showed a significant inverse correlation with serum CRP levels, further supporting its validity as a measure of disease activity and patient well-being. In contrast, no significant associations were found with FCP or Hb, which may suggest that the IBDcQ-8 captures aspects of patient experience that are not fully reflected by these biological markers. An alternative and important explanation for this finding is the high rate of missing data (85%) in fecal calprotectin (FCP) results. In this real-world setting, a significant proportion of patients either did not undergo FCP testing or failed to present their results during their clinic appointments.

 Convergent validity was also supported by the significant correlation between IBDcQ-8 scores and physician-assessed disease activity during the index visit. Moreover, mean scores differed significantly across physician-defined disease categories (remission, partial control, and active disease) using the Kruskal–Wallis test, confirming the instrument’s ability to discriminate between different levels of disease activity.

 The reliability analysis further strengthened the psychometric profile of the questionnaire. Cronbach’s alpha > 0.8 showed that all items in this instrument measure the same underlying construct. Also, the ICC was 0.61 for retesting conducted two weeks later via telephone interviews, indicating acceptable test–retest reliability. However, this result was lower than those reported in previous studies on the IBDcQ, which showed ICCs > 0.9.^11,22,24^ This discrepancy may be attributable to a somewhat lower stability of the instrument in the Persian version compared to the original or other cultural adaptations. An alternative explanation could be the change in administration method from patient self-completion to telephone interview. This is supported by the higher ICC (0.70) observed between telephone interviews conducted one day before or after the first visit and two weeks later. These findings suggest that, although the IBDQ-8 was originally designed as a self-administered tool, it also demonstrates acceptable reliability when administered verbally. This is particularly important for large-scale registries such as the IRCC, where data collection often relies on telephone interviews.

## Conclusion

 The Persian version of the IBDcQ-8 questionnaire demonstrated robust validity and acceptable reliability, supporting its applicability as a standardized tool for evaluating disease-specific quality of life in patients with IBD even when administered by telephone interviews. The availability of a psychometrically sound instrument in the Persian language not only facilitates high-quality research, but also enables clinicians to incorporate PROs into routine practice, thereby enriching patient-centered care. Importantly, the demonstrated reliability and validity ensure that subsequent investigations using this measure, whether in clinical trials, observational studies, or real-world practice, will be grounded in sufficient methodological rigor. Future studies may further explore its responsiveness to change and cross-cultural comparability, consolidating its role as a valuable outcome measure in both national and international research contexts.

## Supplementary File

Supplementary File contains Table S1 and S2.
