# Supplementary material for: Evaluation of the Validity and Reliability of the Persian Version of IBDcQ-8 Questionnaire
Source: Arch Iran Med. 2026 Feb 1;29(2):69–76. doi: 10.34172/aim.36812 (PMC13477566; doi:10.34172/aim.36812)
Supplement: Supplementary file 1 [file aim-29-69-s001.pdf]

Arch Iran Med. February 2026;29(2):69-S1

doi:10.34172/aim.36812

http://journalaim.com

Table S1. Questionnaire for Conducting In-Person or Virtual Interviews to assess content Validity.

**Question Number**	**Completion Date:**	**Interviewee:**							
	**Questionnaire for IRCC**	**Relevance**				**Representativeness**			
		**Verbal Feedback**				**Verbal Feedback**			
	**Section One: Source of Information from the Physician**	**1**	**2**	**3**	**4**	**1**	**2**	**3**	**4**
**1**	Patient's Name:								
**2**	Patient's Surname:								
**3**	National ID Number:								
**4**	Father's Name:								
**5**	Telephone:								
**6**	Mobile Number:								
**7**	Treating Physician's Name:								
**8**	Date of Birth (Solar *Hijri*):								
**9**	Date of Diagnosis: 1. UC; 2. CD; 3. Not Specified								
**10**	If the patient has ulcerative colitis, what has been the extent of involvement?<br>1. Proctitis<br>2. Left-sided colitis<br>3. Pancolitis<br>4. Uncertain								
**11**	If the patient has Crohn's disease, in which part of the gastrointestinal tract is the involvement located?<br>1. Ileal<br>2. Colonic<br>3. Ileocecal<br>4. Other regions								

	5. Uncertain								
**12**	The subsequent questions were examined in a similar manner								

Table S2. Persian Version of IBDcQ-8.

**نمی دانم**	**بله**	**خیر**	
○	○	○	آیا فکر می کنید که بیماری شما در دو هفته اخیر به خوبی کنترل شده است؟
○	○	○	آیا فکر می کنید درمانی که در حال حاضر دریافت می کنید برای کنترل بیماری التهابی روده شما مفید بوده است؟
○	○	○	آیا در دو هفته اخیر هیچگونه از فعالیت های برنامه ریزی شده ی شما به علت علائم بیماری التهابی روده لغو شده است؟
○	○	○	آیا در دو هفته اخیر هیچگاه به علت علائم بیماری التهابی روده از خواب بیدار شده اید؟
○	○	○	آیا در دو هفته اخیر به علت علائم بیماری التهابی روده از درد یا ناراحتی قابل توجه رنج برده اید؟
○	○	○	آیا در دو هفته اخیر اغلب احساس کمبود انرژی و یا خستگی مفرط داشته اید؟
○	○	○	آیا در دو هفته اخیر به علت بیماری التهابی روده احساس افسردگی و یا اضطراب داشته اید؟
○	○	○	آیا در دو هفته اخیر به نظر شما نیاز به تغییر رژیم درمانی دارید؟
